# Including individuals with Parkinson’s disease and deep brain stimulation in rehabilitation trials: feasibility, challenges, and preliminary gait and postural stability results

**DOI:** 10.1016/j.parkreldis.2025.107920

**Published:** 2025-06-12

**Authors:** Anson B. Rosenfeldt, Sara Davidson, Ryan D. Kaya, Elizabeth West, James Y. Liao, Benjamin L. Walter, Hubert Fernandez, Jay L. Alberts

**Affiliations:** a Cleveland Clinic, Lerner Research Institute, Department of Biomedical Engineering, 9500 Euclid Ave., Cleveland, OH, 44195, USA; b Cleveland Clinic, Neurological Institute, Center for Neurological Restoration, 9500 Euclid Ave., Cleveland, OH, 44195, USA

**Keywords:** Parkinson’s disease, Deep brain stimulation, Postural instability, Gait, Dual-task training, Rehabilitation

## Abstract

**Background:**

People with Parkinson’s disease (PwPD) who have undergone deep brain stimulation (DBS) surgery have been historically excluded from rehabilitation clinical trials.

**Objective:**

This project investigated the safety, feasibility, and preliminary efficacy of a dual-task training intervention aimed at improving postural instability and gait dysfunction (PIGD) in PwPD with DBS.

**Methods:**

Symptoms of PIGD were measured with the 2-Minute Walk Test (2MWT) and Timed Up and Go (TUG) test under single- and dual-task conditions.

**Results:**

Five participants completed 97.5 % of the 16 intervention sessions without serious adverse events. One participant was excluded from the motor analysis due to frequent changes in DBS settings. Of the four participants included in the analysis, all demonstrated improvement in the TUG under single- and dual-task conditions. During the 2MWT, two participants experienced an improvement in gait speed under single-task conditions, and three experienced an improvement under dual-task conditions.

**Conclusion:**

Initial data suggest dual-task training is safe and feasible for PwPD with DBS. Having DBS presents unique challenges including advanced motor symptoms, autonomic dysfunction, and potential changes in DBS parameters, that impact intervention delivery, adherence, and outcomes. Despite unique challenges, appropriately selected PwPD with DBS have the capacity to improve PIGD symptoms with dual-task training. To facilitate generalizability across the PD continuum, future rehabilitation trials including PwPD with DBS are justified and recommended.

## 1. Introduction

Since being approved by the FDA approximately 25 years ago, deep brain stimulation (DBS) has become a standard treatment for advanced Parkinson’s disease (PD). An indirect consequence of having DBS is the patient is typically ineligible for a majority of pharmaceutical and rehabilitation clinical trials. The Parkinson’s Study Group (PSG) Functional Neurosurgical Working Group highlighted that most clinical trials, for no abundantly clear reason, list DBS as an exclusion criterion [1]. The lack of inclusion of DBS in PD research was apparent in the 2022 Clinical Practice Guidelines Physical Therapist Management of Parkinson Disease from the American Physical Therapy Association [2], which indicated that no studies meeting established inclusion criteria were available regarding DBS and physical therapy intervention; therefore no specific exercise recommendations were provided for people with PD (PwPD) with DBS. A Delphi consensus panel agreed that physical therapy may help PwPD with DBS, but the quality of evidence supporting exercise intervention in DBS was low; no randomized controlled trials were available [3]. A fundamental gap exists regarding the efficacy of exercise and rehabilitation interventions and the feasibility of including PwPD with DBS in rehabilitation trials.

The benefits of DBS on PD tremor, bradykinesia, and rigidity are well-established; however, the impact of DBS on PD postural instability and gait dysfunction (PIGD) are less clear. Spatiotemporal aspects of gait may improve following DBS; however, some people experience no improvement or even a worsening of postural stability and freezing of gait (FOG) following surgery [4]. Further, initial improvements in PIGD are relatively short-lived, with PIGD declining below pre-surgical levels within two years of surgery [5]. Considering the health and social impacts of falls in PwPD, applying rehabilitation approaches to improve PIGD is necessary to minimize disability in this vulnerable group of patients.

Dual-task (DT) training improves gait in PwPD without DBS [6]. The positive effect of DT training may be centrally mediated, as functional changes in the sensorimotor cortex were present following DT training in PwPD [7]. Assuming the mechanism underlying CNS changes following DT training remains intact in the presence of DBS, there is potential for improvement in those with DBS. The aim of this preliminary investigation was to evaluate the initial efficacy, feasibility, and safety of a DT rehabilitation intervention in PwPD with DBS, and to identify unique challenges associated with enrolling and retaining those with DBS in a rehabilitation intervention.

## 2. Methods

### 2.1. Participants

Inclusion criteria were: idiopathic PD, ability to ambulate for 10+ minutes with or without the use of an assistive device, and implantation of a DBS for the treatment of PD. Exclusion criteria were: medical diagnosis of dementia or other neurocognitive impairment, three or more errors on the Short Portable Mental Status Questionnaire (SPMSQ), severe musculoskeletal or cardiopulmonary issue limiting activity, or other neurological diagnosis. Participants completed the informed consent process approved by the Cleveland Clinic Institutional Review Board prior to participation.

### 2.2. Dual-task intervention

**Fig. 1.**
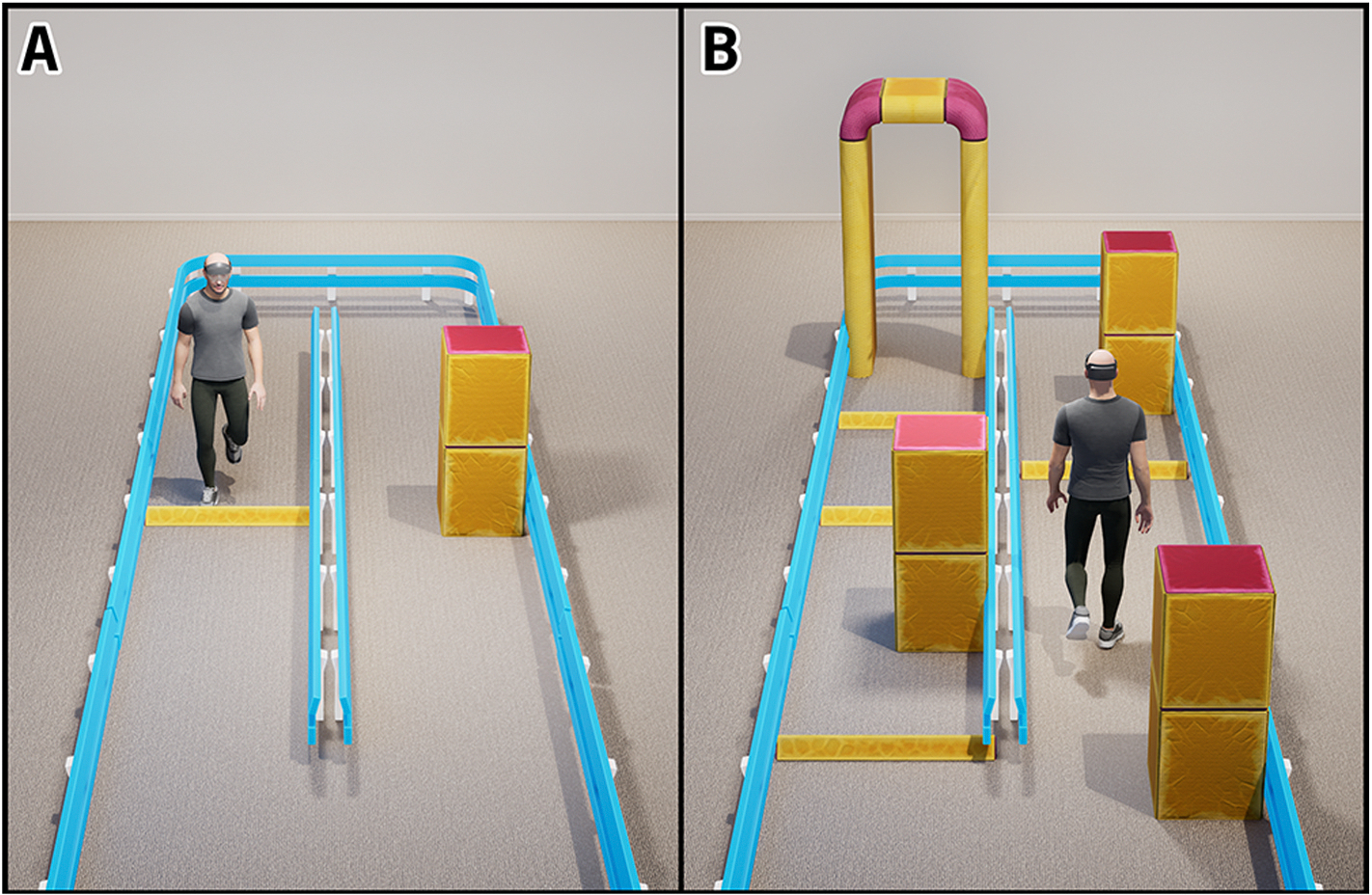
An illustration of an individual wearing the augmented reality headset and performing the DART obstacle course. The user navigates holographic pillars, doorways, and curbs while performing a secondary cognitive task. The digital obstacles are only visible to the user wearing the augmented reality headset. The environment in (A) is considered simple with only two obstacles while (B) represents a complex obstacle course. In addition to modifying the physical task to meet the individual needs of the user, the difficulty of the cognitive task can be adjusted.

The Dual-Task Augmented Reality Treatment (DART) platform, a validated and effective digital therapeutic for DT training in PD [6], was used to deliver the intervention. The DART platform uses a head-mounted augmented reality (AR) device (HoloLens2 (HL2), Microsoft Corporation, USA) to place digital holograms in the physical environment of the user. The holograms create interactive environments that target patient-specific mobility impairments (Fig. 1). For each DART module, a motor task is paired with a cognitive task that addresses attention, memory, language, or executive function. Participants are guided through each session by a digital avatar. The avatar provides auditory instructions and visual demonstration of task requirements. DART modules are self-directed, and the participants can repeat the instructions to ensure task understanding.

For this project, a physical therapist programmed 7–10 DT modules to create an interventional session consisting of 30 min of active DT training over a 45–60 min session. The HL2, with its accelerometer and gyroscope, provided objective outcomes (e.g., gait speed, step length, and turn duration) for each module. The difficulty of rehabilitation sessions was increased or decreased by the physical therapist based on participant performance in the prior session. A total of 16 sessions were performed over eight weeks in the ON antiparkinsonian medication and ON DBS condition.

### 2.3. Outcomes

Outcomes were gathered at baseline and end of treatment while ON medication and ON DBS by a study team member blind to study design. The 2-Minute Walk Test (2MWT; distance ambulated over 2-min) and instrumented Timed Up and Go (TUG; transfer from chair, 3 m ambulation, turn, and return to chair) were evaluated under single-task (ST) and DT conditions. During DT conditions, the Serial 7’s task was performed. To negate practice or learning effects, the Serial 7’s task was not part of interventional sessions.

Feasibility was evaluated by participant recruitment and retention, adverse events, intervention adherence, and Systems Usability Scale (SUS) [8].

## 3. Results

Nine individuals with bilateral STN-DBS were screened for participation; seven met inclusion/exclusion criteria and were enrolled. All enrolled participants had bilateral subthalamic nucleus DBS. Mean (range) demographics were as follows: age 67 (52–76) years, disease duration 16.1 (10–22) years, DBS placement 6.1 (1–14.25) years prior, falls in the previous 6 months 18 (1–72), MDS-UPDRS III in the ON medication/ON DBS condition 40 (25–62) points, and LEDD 427 (300–600) mg. Six of the seven were classified as Hoehn & Yahr III; one was Hoehn & Yahr II. Two participants withdrew (severe orthostatic hypotension and a loss of interest), leaving five that completed the intervention. Those five completed 97.5 % of rehabilitation sessions. One participant experienced frequent adjustments in DBS settings (4–5 changes over the 16-session intervention) resulting in variability of motor symptoms. The participant’s feasibility data, but not motor, were included in the analysis.

### 3.1. Motor outcomes

**Fig. 2.**
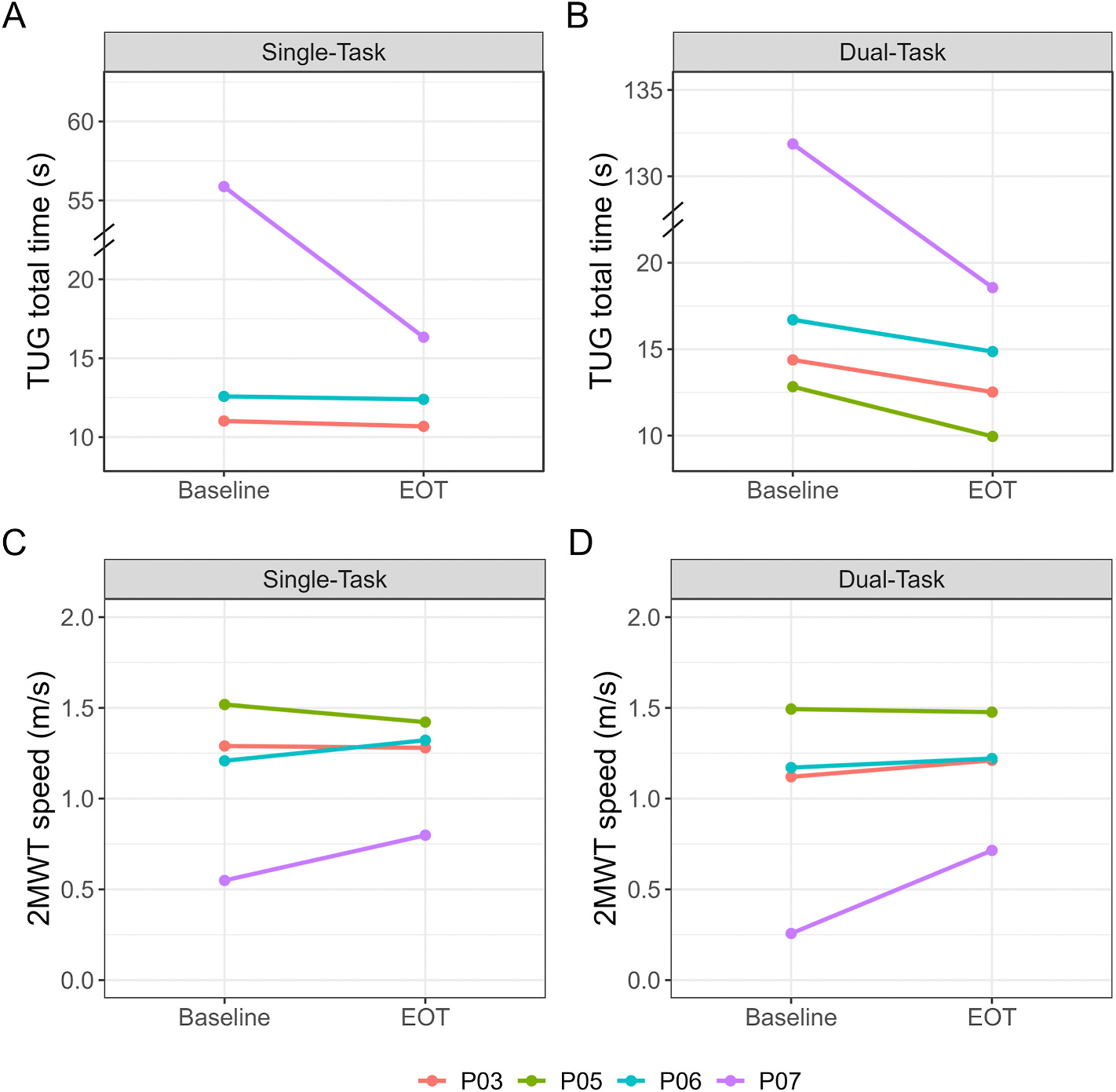
Changes from Baseline to End of Treatment (EOT) for each participant. **(A&B) Timed Up and Go (TUG).** All participants improved (decreased) the time to complete the TUG under single- and dual-task conditions. *One participant (P05) did not have single-task TUG time due to a technology error. **(C&D) 2-Minute Walk Test (2MWT).** Two of the four participants improved (increased) gait speed during the 2MWT under single-task conditions; three of the four participants improved under dual-task conditions.

Individual performance for the TUG and 2MWT under ST and DT are provided in Fig. 2; one participant did not have a ST TUG time due to a technology error. Functional mobility measured by the TUG improved for all participants under ST and DT conditions following the DART intervention. Under ST conditions, P03 and P06 had small improvements in their TUG time (<0.5 s), while P07 had a nearly 40 s improvement. There was greater overall improvement in TUG times under DT conditions, with all four participants decreasing overall TUG time. P03, P05, and P06 all improved their overall TUG time by ~2–3 s, and P07 demonstrated the largest change with an improvement of 113 s. The TUG performance for P007 improved substantially as the intervention reduced the frequency and duration of FOG episodes.

During the 2MWT, two of the four participants increased gait speed by 0.11 m/s (P06) and 0.25 m/s (P07) under ST conditions while P03 gait speed remained consistent and P05’s gait speed decreased by 0.10 m/s. Under DT conditions, three of the four participants increased their gait speed by 0.09 m/s (P03), 0.05 m/s (P06), and 0.46 m/s (P07); P05 did not change.

### 3.2. Adverse events

No serious adverse events occurred over the course of the intervention. Two non-serious adverse events occurred; one participant experienced an exacerbation of existing sciatic nerve pain while one individual experienced a non-injurious fall during a rehabilitation session due to a FOG episode. Notably, the participant who experienced a fall reported multiple FOG-related falls per week at baseline.

### 3.3. Usability and satisfaction

Mean SUS score was 83.0 (11.4) which is classified as excellent in terms of platform usability. [8].

## 4. Discussion

Despite advances in DBS technology, it does not arrest the neurodegenerative process of PD. Historically, excluding PwPD with DBS from rehabilitation clinical trials was defensible due to concerns of response variability or protocol adherence. The current data, albeit initial, suggest that PwPD with DBS can complete a challenging DT rehabilitation paradigm and experience improvements in aspects of physical mobility. The feasibility, safety, and preliminary efficacy data are consistent with a recent rehabilitation trial in a similar cohort of PwPD with DBS [9]. Overall, the preliminary motor data presented in this report support moving forward with a larger clinical trial in PwPD with DBS.

Prior to the initiation of larger rehabilitation trials in PwPD with DBS, specific considerations regarding recruitment and assessment are necessary. One individual withdrew from the study due to severe orthostatic hypotension rendering them unable to tolerate prolonged standing and walking. Autonomic dysfunction should be evaluated as part of the screening process and contemplated as an exclusion criterion. Another participant was not included in the outcome analyses as their DBS parameters were not clinically stable despite having been implanted 12 months prior. Stability of DBS, independent of duration of implantation, should be considered when enrolling participants. A 2023 meta-analysis evaluating the efficacy of DT training in PD reported that falling was the most prevalent adverse event reported [10], and indeed one of the participants with a history of frequent falls in this trial experienced a fall during training. The risk of falling should not preclude a patient from being enrolled and completing a mobility intervention, especially one that is intended to reduce PIGD symptoms. Rather, precautions such as guarding, use of a gait belt, and appropriate exercise prescription should be implemented to mitigate the risk of falls. Appropriate inclusion/exclusion criteria are likely to improve recruitment and retention above what was observed in this pilot project.

The seven enrolled participants had a disease duration of 17.0 (range 10–23) years and six of seven enrolled were H&Y III, indicating significant PIGD symptoms. Comparatively, a recent systematic review and meta-analysis from the Cochrane Library reporting on 154 randomized controlled PD exercise trials reported mean disease ranging from 0.3 to 13.3 years with several studies excluding H&Y III or greater [11]. Despite advanced symptoms and lengthy disease durations, all participants improved in at least one aspect of functional mobility and gait. While some of the participants (P06 and P07) improved in all motor outcomes, others (P03 and P04) improved in some. The variability of responses is not unusual in rehabilitation trials and highlights the importance of contemplating potential response variability when powering future RCTs. It is possible that improvements in the current study may have been greater with an increased dose (i.e. duration or frequency) of rehabilitation.

Strong adherence, lack of serious adverse events, and excellent usability ratings on the SUS were comparable to our previous DART RCT with mild to moderate PwPD without DBS [6]. The DART platform was created using the Develop with Clinical Intent (DCI) model [12] with consideration that older adults with neurological disease were the end-users. Specific PD- and age-related cognitive and motor declines were considered throughout the development process and resulted in a user experience and interface in which participants could listen to the directions multiple times. A key element of any intervention, is ensuring the activity and technology consider the capabilities of the end-user during the design phase.

Results from rehabilitation and exercise-based RCTs interventions show promise in improving PD related symptoms and overall quality of life; unfortunately, the results of these trials cannot be generalized to PwPD with DBS. This pilot project indicates that DT training in PwPD with DBS is safe, feasible, and has the potential to improve PIGD. Executing an interventional trial in a DBS population comes with unique challenges such as advanced motor symptoms including falls, autonomic dysfunction, and alterations in DBS settings that acutely impact symptoms. Future RCTs addressing the impact of exercise and rehabilitation protocols on PwPD with DBS are recommended. Trial options include stand-alone protocols for PwPD with DBS or including PwPD with and without DBS in larger RCTs. In the design and execution of the latter option, a priori or post-hoc subgroup analyses can be completed to determine if the treatment effect is present or different for those with DBS. Undergoing DBS should not be considered the final attempt or viewed as the Hail Mary at improving PD patients’ overall quality of life and mobility; targeted rehabilitation protocols have the potential to augment DBS and medical treatment.

## Data Availability

The data that support the findings of this study are available from the corresponding author upon reasonable request.
